# Orthopedic workforce planning in Germany – an analysis of orthopedic accessibility

**DOI:** 10.1371/journal.pone.0171747

**Published:** 2017-02-08

**Authors:** Jan Bauer, Peter Müller, Werner Maier, David A. Groneberg

**Affiliations:** 1 Institute of Occupational, Social and Environmental Medicine, Goethe University, Germany; 2 Public Health Foundation (‘Stiftung Gesundheit’), Hamburg, Germany; 3 Institute of Health Economics and Health Care Management, Helmholtz Zentrum München, German Research Center for Environmental Health (GmbH), Ingolstädter Landstr. 1, Neuherberg, Germany; Peking University, CHINA

## Abstract

In Germany, orthopedic workforce planning relies on population-to-provider-ratios represented by the ‘official degree of care provision’. However, with geographic information systems (GIS), more sophisticated measurements are available. By utilizing GIS-based technologies we analyzed the current state of demand and supply of the orthopedic workforce in Germany (orthopedic accessibility) with the integrated Floating Catchment Area method. The analysis of n = 153,352,220 distances revealed significant geographical variations on national scale: 5,617,595 people (6.9% of total population) lived in an area with significant low orthopedic accessibility (average z-score = -4.0), whereas 31,748,161 people (39.0% of total population) lived in an area with significant high orthopedic accessibility (average z-score = 8.0). Accessibility was positively correlated with the degree of urbanization (r = 0.49; p<0.001) and the official degree of care provision (r = 0.33; p<0.001) and negatively correlated with regional social deprivation (r = -0.47; p<0.001). Despite advantages of simpler measures regarding implementation and acceptance in health policy, more sophisticated measures of accessibility have the potential to reduce costs as well as improve health care. With this study, significant geographical variations were revealed that show the need to reduce oversupply in less deprived urban areas in order to enable adequate care in more deprived rural areas.

## Introduction

Orthopedics play an important role as health care providers both in hospitals and in practices. Access to their specialist input can support primary care management of various diseases such as osteoarthritis or musculoskeletal disorders [1–3]. Furthermore, there have been geographical variations shown for primary hip and knee joint replacement, which is often related to osteoarthritis [4]. These geographical variations could be related to a differing access to orthopedic care [5]. However, valid data regarding access to orthopedic care in Germany are lacking. Generally, access to health care providers is of increasing interest in scientific literature [6,7]. However, access often lacks an appropriate definition due to its multi-dimensional structure. Five dimensions of access have been described: availability, accessibility, accommodation, affordability and acceptability [8]. Availability (number of health care providers) and accessibility (i.e. the distance/time from demand to supply of health care) as spatial factors are referred to as ‘spatial accessibility’ [9]. So far, the scientific literature regarding access to orthopedic care has mainly focused on ‘obtaining an appointment’ [5,10,11]. Here, the insurance status of patients has been shown to have a significant effect on the appointment rate for both adults and children.

In general, the distribution of orthopedics is regulated in Germany. In order to allocate the orthopedic workforce, a simple physician-to-population ratio (PPR) is used: A PPR of 1:14,101 in large urban areas and 1:26,712 in more dispersed areas represents a service provision degree of 100% (i.e. the population is per definition fully provided with orthopedic health care) [12]. An excess of more than 10% is considered an oversupply [12]. However, PPRs as planning tools have strong limitations in regard of being a robust and valid accessibility measure [13]. PPRs represent a simple method that measures spatial accessibility by the supply–demand match ratio in an area [14]. However, PPRs don`t reveal detailed spatial variations within an area nor account for boundary crossing of patients and physicians. Further measures of spatial accessibility include 1) the distance to the nearest provider, 2) the average distance to a set of providers and 3) gravity-based models [9]. However, while the first is insensitive for congestive areas with more than one provider, the second overweighs providers peripherally located. Finally, gravity models, as first suggested by Joseph et al., address these limitation by considering interactions between patients and physicians across boundaries [15]. Floating Catchment Area (FCA) methods represent a special case of gravity models and are popular measures of spatial accessibility [16–18]. Due to major limitation of other accessibility measures as explained above, we used the FCA approach.

The scarce literature of orthopedic workforce analysis in Germany has focused on PPRs or examined non-spatial factors [4,19]. Therefore, our objective was to provide a nationwide analysis of orthopedic accessibility in Germany using a sophisticated and robust assessment. In particular, we aimed at answering the following important questions for patients, orthopedics and health care planners: 1) Does orthopedic accessibility vary geographically on a national scale in Germany? 2) Is there an urban-rural, intra-urban or social gradient present? 3) Is the current orthopedic workforce planning adequately considering orthopedic accessibility?

## Materials and methods

### Geospatial methods

We used the integrated Floating Catchment Area (iFCA) method to measure spatial accessibility [18]. This method is based on the Two Step Floating Catchment Area (2SFCA) method [16]. However, the iFCA method addresses limitations of the original 2SFCA method regarding catchment sizes, distance decay and competition parameters by integrating improvements, which have been proposed by recent literature: Luo et al. introduced variable instead of fixed catchment sizes [20]. They further developed the enhanced (E)2SFCA method by dividing catchment areas into three zones and applying a discrete Gaussian function as the decay function [17]. Regarding the distance decay, several other decay functions have been used: 1) gravity function, 2) Gaussian function, 3) binary discrete, 4) multiple discrete, 5) kernel density, 6) three zone hybrid and 7) logistic based functions [14,21]. Competition in the demand-supply system of healthcare has been acknowledged by integrating an additional variable based on competition by accounting for the number of competitors within a catchment [22] and by integrating the Huff Model into the 2SFCA method [23]. Finally, the iFCA method integrated the above mentioned earlier improvements by using variable catchment sizes, a logistic based decay functions and the Huff Model. The resulting formula has the following form:

$$AI_{x}=\sum_{y\in \left(d_{xy}\leq C_{x}\right)}\frac{S_{y}∙f_{adj}\left(d_{xy}\right)∙f_{con}\left(d_{xy}\right)}{\sum_{x\in \left(d_{xy}\leq C_{x}\right)}P_{x}∙f_{adj}(d_{xy})∙Prob_{demand}}$$ (1)

*AI*_*x*_ is the accessibility index at location *x*. *S*_*y*_ is the capacity of orthopedics (headcount) at location *y* (i.e. the practice), *P*_*x*_ is the population size at population location *x* (i.e. the grid cell centroids). Within accessibility measures, especially distance based conceptualizations of access (in minutes or kilometers) have been used [24,25]. Accordingly, all distances (in minutes) between *x* and *y* (*d*_*xy*_) were calculated for a predefined global catchment size of *C*_*glob*_ = 60min by car on a road network. *f*_*adj*_*(d*_*xy*_*)* is the adjusted and *f*_*con*_*(d*_*xy*_*)* the constant distance decay function. *f*_*adj*_*(d*_*xy*_*)* is adjusted to the distance distribution (median and standard deviation (SD)) of the first 50 orthopedic practices (only counting practices not headcounts) for each population location *x*, which results in differently shaped functions for each location *x*. McGrail et al. used the distance to the first 100 primary care services as a cut-off in their analysis to model rural-urban distinctions of catchments [26]. Since orthopedic care is more scarce than primary care in Germany, we used 50 practices as the cut-off. Furthermore, by using n = 50 as the cut-off, the overall mean distance to 50 practices in our study sample was 30min and therefore half of the global catchment size of 60min. *f*_*con*_*(d*_*xy*_*)* is identical for all population locations and solely depends on the global catchment size. Both functions generate weight values dependent on the distance between practice and population locations and are based on the cumulative distribution function of the logistic function (downward sigmoid function). The combination of both functions results in an individual effective catchment size *C*_*x*_ for each population location *x*. C_x_ is defined as the distance *d* for which *f*_*adj*_*(d)·f*_*con*_*(d)* = 0.01. The competitor based probability of demand is represented by *Prob*_*demand*_ (see Huff Model [23]).

Basically, two steps have to be performed within this method: First, for each orthopedic practice location y, the workforce supply (headcount) is divided by the cumulative demand (denominator) that is put on the practice by all population locations x in whose catchment C_x_ the practice is located. Hereby a physician-to-population ratio (PPR) is computed. Second, for each population location x, all PPRs (accounted for distance decay) within the catchment C_x_ are summed up. Hereby the Accessibility Index AI_x_ is computed. High AI values represent areas with high spatial accessibility whereas low AI values represent areas with low spatial accessibility. However, since benchmark data are lacking the AI values must be considered as relative measures. We further aggregated AI values on higher administrative area levels similar to the 3-Step approach introduced by Bell et al. [27,28]: We averaged accessibility indices of all grid cells whose centroid fell into the administrative area boundary.

**Fig 1:**
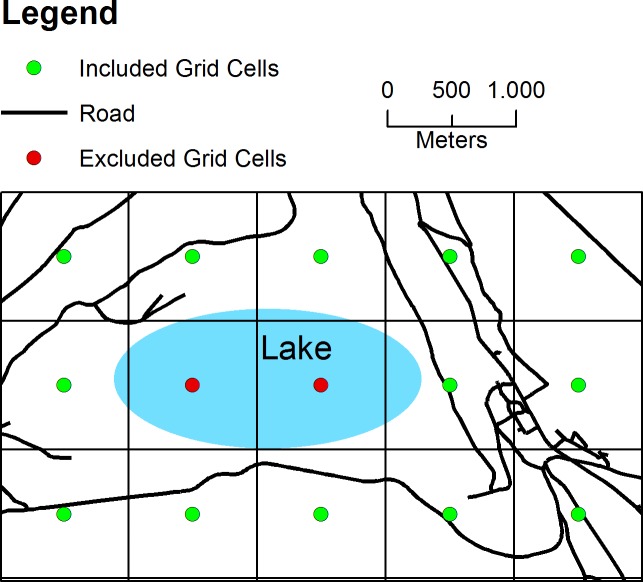
Schematic example of the population allocation process using grid cells in a municipality comprising n = 15 grid cells and a population size of n = 12,000. Further information are provided within the text.

For the analysis, we subdivided Germany in km^2^ grid cells (n = 357,711). A special focus was put on German cities with a population of more than one million (n = 4). These metropolises were additionally subdivided in hectare grid cells (n = 235,548). Further, we excluded all grid cells whose centroid was >500m (for hectare grid cells >50m) away from a road that was accessible by car. Hereby, non-accessible areas were excluded (e.g. rivers, lakes). We proportionally allocated population sizes to each grid cell centroid according to their number in the respective administrative area. In Fig 1 we provided an example of the population allocation process in an area of 15km^2^ (i.e. 15 grid cells) representing a municipality with a population size of n = 12,000.

**Fig 2:**
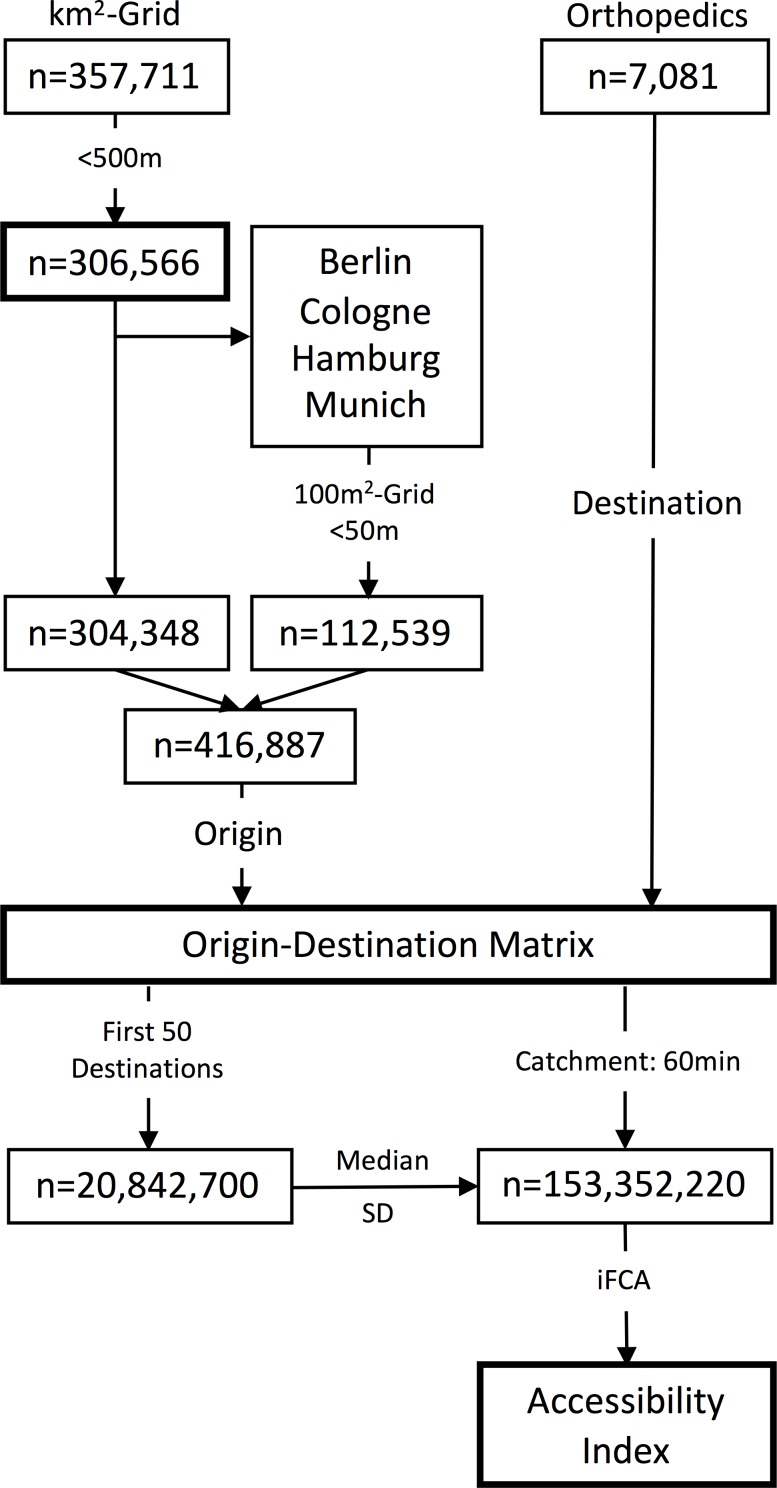
Data preparation workflow. SD: standard deviation; min: minutes; iFCA: integrated floating catchment area method.

Due to the lake in the middle of this municipality, there are two grid cells, whose centroid is more than 500m away from the next road. Therefore, these two grid cells were excluded. The population size was then allocated to the remaining 12 grid cells within this municipality meaning that each grid cell has been allocated a population size of n = 1,000. See also Fig 2 for the data preparation workflow.

The origin-destination matrix resulted in n = 153,352,220 distances, which were used for the computation of the accessibility index (AI).

### Data sources

Demographic data: All population data were from 2014. For the metropolises, data were retrieved from official communal administrative statistics. Population data of municipalities were obtained from the Federal Statistical Office in Germany [29].

Geographic data: Geographic data of metropolises were retrieved from local authorities. Geographic data of municipalities (n = 11,299) and districts (n = 402) were retrieved from the Federal Agency for Cartography and Geodesy as of 2016 [30]. Furthermore, geographic data of the official planning regions as defined by the Federal Joint Committee were generated as of 2013 (n = 385) [12].

Road Network: Road network data of Germany were obtained from TomTom Multinet data (TomTom N.V., Amsterdam, Netherlands) as of 2016.

Data of orthopedics: Practice locations were retrieved from the ‘Public Health Foundation’ (‘Stiftung Gesundheit’, Hamburg, Germany) as of 2015 [31]. Furthermore, the official degree of care provision (in %) of orthopedic care in Germany (on planning region level as of 2014) was retrieved from the National Association of Statutory Health Insurance Physicians [32]. This degree of care provision represents the current planning tool of orthopedic care in Germany.

### Data enrichment on municipality level

For the measure of urbanity we used the degree of urbanization (DEGURBA) as defined by EUROSTAT (as of 2015) [33]. Furthermore, the classification of major urban areas as defined by the Federal Office for Building and Regional Planning in Germany (as of 2013) was used [34]. In addition, we enriched municipality data with regional deprivation data using the German Index of Multiple Deprivation (GIMD) as of 2010 [35,36].

### Statistics

All spatial calculations and data preparations were done with ArcGIS 10.4 and ArcGIS Pro 1.2 (ESRI Inc., Redlands, USA). Based on the Getis-Ord Gi* statistic, a hot spot analysis was performed. Further statistical calculations (including standardization using z-scores) were performed with SPSS Version 23 (IBM, Armonk, USA). We used non-parametric testing to test for significant differences (Kruskal-Wallis-Test) and correlations (Spearman’s Rho, two tailed). In addition, the calculation of catchment sizes was performed with RStudio (R Core Team, Vienna, Austria) including the packages ‘rootSolve’ and ‘plyr’.

## Results

**Fig 3:**
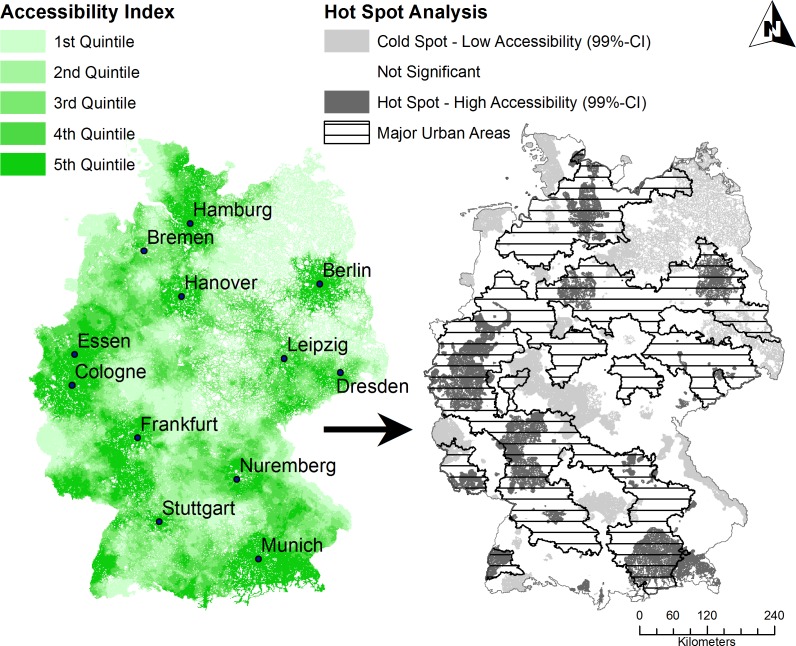
Spatial accessibility of orthopedics in Germany (km2-Grid) with Hot Spot Analysis. CI: confidence interval.

Visualization of orthopedic accessibility on national scale showed geographical variations throughout Germany (Fig 3).

Higher accessibility was present in or around major cities and lower accessibility was present in more rural areas, especially in North East and Central Germany. Varying spatial accessibility was further supported by the descriptive results, which revealed a mean accessibility index of 0.0027 (SD: 0.0019) with a range of 0–0.0558. The mean distance to the first orthopedic practice on national scale was 10.5min (SD: 5.3min). It has to be noted that 52 out of 416,887 population locations could not reach an orthopedic within 60min (AI = 0). The mean effective catchment size was 39.2min (SD: 9.7) with a range of 15-60min. Therefore, the effective catchment also showed significant geographical variations, mainly depending on the degree of urbanization with a negative correlation of r = -0.67 (p<0.001). In other words, the more urban the area, the smaller was the effective catchment size. Furthermore, assuming an identical need for orthopedic care among the German population, the iFCA method calculated an average potential patient number per practice of 7,153 (SD: 2,523).

In addition, we performed a hot spot analysis, which further supported the visual findings reported above (see also Fig 3): In terms of population, 5,617,595 people (6.9% of total population) lived in an area (60,430km^2^) with significantly low orthopedic accessibility (average z-score: -4.0), whereas 31,748,161 (39.0% of total population) lived in an area (43,577km^2^) with significantly high orthopedic accessibility (average z-score: 8.0).

For benchmark purposes we compared the iFCA method with earlier measurements from this family: There was a positive correlation of r = 0.57 (2SFCA) and r = 0.64 (E2SFCA).

### Urban vs. rural

Orthopedic accessibility in Germany was significantly related to the degree of urbanization: There was a positive correlation with the degree of urbanization (DEGURBA) of r = 0.32 (p<0.001). In other words, in densely populated areas orthopedic accessibility was significantly higher than in thinly populated areas (AI: 0.0047 vs. 0.0023; p<0.001). However, taking the classification of major urban areas as provided by the Federal Office for Building and Regional Planning into account, the correlation with urbanity increased to r = 0.49 (p<0.001). The extent of these major urban areas is shown in Fig 3. Furthermore, there were significant differences among the five classes of major urban areas in Germany (p<0.001): Orthopedic accessibility was significantly lower outside of major urban areas and constantly increased towards the center of the major urban area (AI: 0.0052 vs. 0.0020; see Table 1).

**Table 1:** Orthopedic spatial accessibility in major urban areas in Germany. Subdivisions of major urban areas (e.g. ‘narrow urban’) as defined by the Federal Office for Building and Regional Planning in Germany [23]. SD: standard deviation.

	Germany	Major Urban Area				Outside Major Urban Areas
		Center	Central Buffer	Narrow Urban	Wide Urban	
**Population (n)**	81,368,029	23,601,177	12,798,972	13,120,651	11,520,753	20,326,476
**Area (km**^**2**^**)**	306,566	11,424	13,078	53,914	79,301	148,849
**Accessibility Index**						
Mean	0.0027	0.0052	0.0045	0.0034	0.0027	0.0020
SD	0.0019	0.0056	0.0023	0.0019	0.0014	0.0009

In addition, the aforementioned areas can further be divided into 266 different regions. As suggested by the varying accessibility indices among the five classes (especially regarding the ‘centers’), significant differences were present among these 266 regions: The highest accessibility index was present in Munich with AI = 0.0080 (SD = 0.0054), whereas the lowest was present in Torgelow-Ferdinandshof with AI = 0.0003 (SD: 0.0001). In the supporting information file 1 (S1 Appendix) we provided detailed results for all 266 regions in Germany.

**Fig 4:**
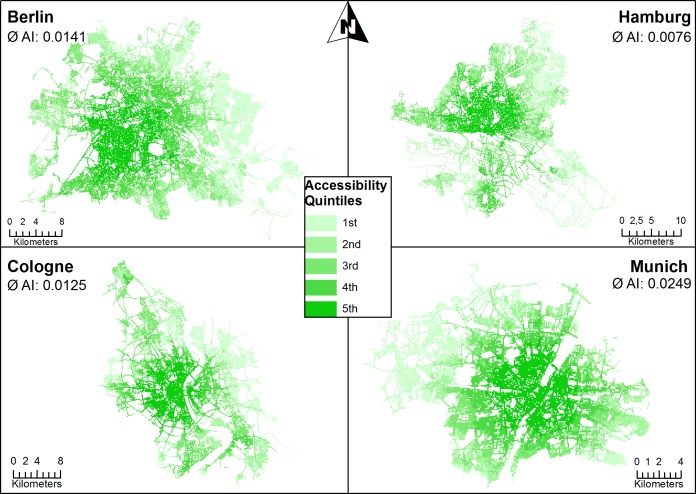
Orthopedic spatial accessibility in major cities (hectare grid cells) in relation to the city average. ∅ AI: mean accessibility index.

We further focused on the four largest cities in Germany: Berlin, Cologne, Hamburg, and Munich (see Fig 4).

It has to be noted that the extent of the urban areas reported above were different from city boundaries (i.e. the urban area ‘Munich’ represents an area of 8,629km^2^, whereas the city ‘Munich’ represents an area of 311km^2^).

There was a clear centripetal pattern of higher orthopedic accessibility towards the geographic city center in all four cities. Furthermore, lower accessibility was present in the East of Berlin and West of Munich. However, since mean accessibility indices ranged between 0.0076 in Hamburg and 0.0249 in Munich, it has to be noted that compared to the rural areas in Germany, even the lowest accessibility index (AI = 0.0032) was still higher than the average accessibility index in Germany (AI = 0.027). Therefore, low and high orthopedic accessibility displayed in Fig 4 are relative measures in regard to the city average.

### Orthopedic accessibility and area deprivation

The analysis on municipality level revealed a significant negative correlation with the overall GIMD of r = -0.47 (p<0.001). In other words, the higher the orthopedic accessibility, the less deprived was the area. Among the different domains comprising the GIMD, the income domain showed the highest correlation with r = -0.49 (p<0.001). Therefore, a significant area-level social gradient was present regarding orthopedic accessibility.

### Orthopedic accessibility in planning regions

**Fig 5:**
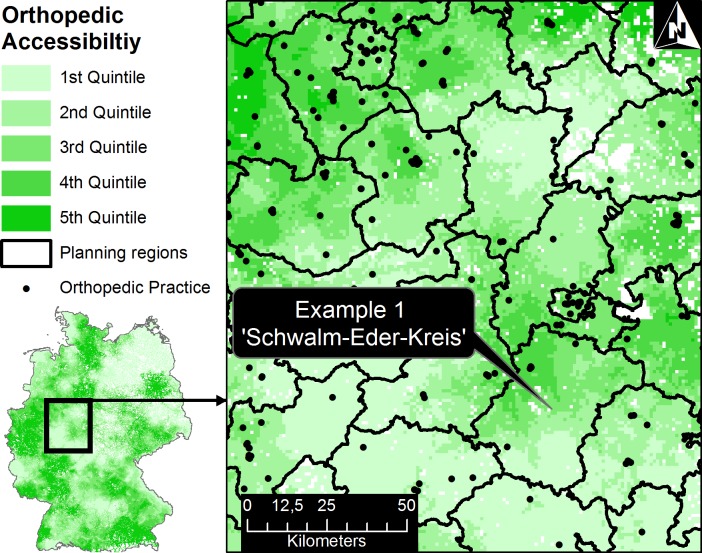
Large scale map to demonstrate geographic variations of orthopedic accessibility within planning regions.

Boundaries of planning regions in Germany are regularly adjusted. However, the current planning regions as of 2014 (n = 385) do not reflect small area variations in Germany. As seen in Fig 5, there are substantial differences regarding orthopedic accessibility within planning regions.

The average difference of the accessibility index (maximum—minimum) in each planning region was 0.0038 (SD: 0.0042). In the example ‘Schwalm-Eder-Kreis’ in Fig 5 the range of orthopedic accessibility was 0.0006 up to 0.0032. Therefore, accessibility varied significantly within planning regions.

In addition, we aggregated accessibility indices on planning region level to compare spatial accessibility with the official degree of care provision. In order to make both comparable, we computed z-scores and calculated z-score differences. On the one hand, orthopedic spatial accessibility was positively correlated with the official degree of care provision (r = 0.33; p<0.001). For example, in the planning regions ‘Tübingen’ and ‘Karlsruhe’ the difference between z-scores were not significant (difference of z-scores: -0.02 and 0.03). On the other hand, there were also distinct differences throughout Germany: The degree of care provision underestimated orthopedic service availability and accessibility especially in major cities such as Berlin, Cologne, or Munich (difference of z-scores: 5.20, 8.69 and 4.93). In other words, the supply of orthopedic workforce was actually higher than current workforce planning suggested. Furthermore, it overestimated orthopedic service availability and accessibility for example in ‘Konstanz’ (difference of z-scores: -3.0) or ‘Zwickau’ (difference of z-scores: -3.1), where the supply of orthopedic workforce was actually lower than the current workforce planning suggested. However, no clear pattern can be extracted that could explain the revealed differences. We provided detailed results for all 385 planning regions in the supporting information file 2 (S2 Appendix).

## Discussion

With this high resolution analysis of orthopedic accessibility in Germany, significant geographical variations could be revealed on national scale. We were able to demonstrate a clear urban-rural, intra-urban and social gradient regarding orthopedic accessibility. Furthermore, we could demonstrate deficiencies of the current official workforce planning tool while highlighting benefits of more sophisticated tools such as the iFCA method. These results can be used by health policy makers to exactly determine where to start reallocating processes in order to optimize the delivery of orthopedic care in Germany. Optimization should start by reducing oversupply in urban areas and using these resources to reduce undersupply in more rural and socially deprived areas. However, an urban-rural gradient was expected since it is known for the general practitioner (GP) workforce in Europe as well as for the orthopedic workforce in the United States [37–40]. However, the extent of the disparities presented in this article must alarm policy makers to balance mismatched workforce in Germany. It has to be noted that balanced care cannot mean the same amount of care for all areas. With limited resources, rationalizing has to be implemented. Therefore, areas have to be identified that could be adequately cared for with less resources. As shown by our results such areas mostly represent less deprived urban areas. Furthermore, such areas can also be identified even within urban areas themselves, which is in line with current literature [25]. In addition, prioritization of orthopedic resources must also be implemented. However, in order to prioritize, population data have to be enriched with actual clinical data allowing to estimate the clinical need for orthopedic care. Therefore, future workforce planning should integrate clinical data on regional level.

In 2014 the average official degree of orthopedic care provision in Germany (in percent of optimal PPR) was 139.0%. Only four planning regions had less than 100% [32]. By definition, the majority of planning regions was oversupplied (>110%). But it has to be noted that the PPR used for health care planning in Germany can be adjusted to accommodate several factors including demographic, socioeconomic and geographic factors (see § 2 BPL-RL). However, these adjusting factors are defined individually on regional level. Therefore, their application can significantly vary on national level. These aspects limit the comparison with orthopedic accessibility, as measured in this study. However, the degree of care provision was positively correlated with orthopedic accessibility, which partially supports the current planning tool. Still, due to major limitations of PPRs, the validity of the current planning tool has to be questioned [13].

Health care services discrepancies, as presented above within the orthopedic care in Germany, are also present within other types of the health care associated services. As reported by Jones et al. there are differences in access to dental services with an urban clustering of dental care [41]. Furthermore Engler-Stringer et al. have shown that such disparities can even be revealed in the access to healthy food [42]. However, since orthopedic diseases are likely to interfere with the patients’ physical mobility, accessibility of orthopedic care is of utter importance. Functional limitations of patients (e.g. elderly or wheel chair patients) have been shown to significantly influence access to orthopedic care [19,43]. Therefore, adequate access to orthopedic care must be considered even more important compared to other medical specialties such as gynecology, urology or dermatology, where physical limitations only play a minor role from the medical point of view. Due to its major impact on accessibility, physical accessibility of orthopedic services needs to be further evaluated. Therefore, future research should focus on physical barriers compromising adequate access to orthopedic care.

In a study in Ontario, orthopedic accessibility was measured by a gravity model, which is conceptually similar to the method used in our study [25]. The catchment used for the gravity model was 50km. However, distances were measured using the Minkowski metric and not actual road distances as in our study [44]. The reported median distance to the nearest orthopedic surgery in Ontario was 11.5km (range: 1-342km). In our study the average distance (in minutes) to the nearest orthopedic surgery was 10.5min and a global catchment of 60min was used. Froelich et al. reported average 1-way distances traveled by patients to orthopedic outpatient clinics of 21.3–36.2 miles with significant differences between the insurance status (Medicaid: 36.2 miles; Private insurance: 24.1 miles) [24]. However, distances based on minutes have to be considered more realistic than distances based on kilometers [45]. Furthermore, the travel mode including commuting behavior influences accessibility [46]. However, no direct health effects were shown for commuting [47]. The reported influence of the insurance status on access to orthopedics has been mainly analyzed with the focus on ‘obtaining an appointment’: Statutory insured patients had more difficulties obtaining an appointment at orthopedic surgeries than privately insured patients [5,10,11]. Therefore, socioeconomic differences play an important role in health care access [48]. Further studies even suggested a link between the geographical aspect of accessibility and the socioeconomic status [49]. In our study a clear area-level social gradient was revealed with better accessibility for the less deprived population. However, it has to be noted that orthopedic accessibility as conceptualized in this study explicitly excluded social factors.

Further evidence of significant differences regarding access to health care in major cities is provided by Mullen et al.: The authors reported significant differences in access to primary stroke centers based on ethnicity in major cities across the United States: 89% of the population in major cities were able to access a primary stroke centers within 60min compared to 1% in rural areas [50]. This underlines the greater significance of differences in access to health care between rural and urban areas, than within an urban area itself [40,51]. These findings are in line with our result of significant urban-rural differences of orthopedic accessibility in Germany. For gynecology and obstetrics in Germany a centralization of care was reported to be possible without compromising comprehensive access [52]. However, it remains unclear if this can be extrapolated to orthopedic care.

It has to be noted that spatial accessibility represents the potential access in contrast to the actual used access. Therefore, reported results are limited to the potential access. Furthermore, no threshold values of orthopedic accessibility are available to distinguish poor access from good access. Therefore, the results mainly represent relative measures. More studies should be conducted to evaluate and establish absolute threshold values of spatial accessibility. Furthermore, accessibility highly depends on the definition of access. We used the definition of spatial accessibility by Guagliardo et al. and operationalized by Luo et al., which overcomes several limitations of earlier measurements and therefore represents an adequate measurement [9,16]. Still, this method has limitations, especially regarding the adequate catchment size and the appropriate distance decay function [14]. However, the positive correlation of the iFCA method with earlier methods from the floating catchment family (2SFCA and E2SFCA) supported the validity of the iFCA method.

Another factor influencing spatial accessibility and its measurement is the travel mode: A survey among n = 1061 residents in Berlin revealed that the majority of patients (59%) reached their primary care physicians via walking [53]. In regard to specialized physicians, 47–67% used a car or public transit as the mode of transportation. In our analyses we used the travel time by car as the distance measurement and therefore our results are limited to the availability and usability of a car. Furthermore, only orthopedic practices were included, despite the fact that the coordination of both the hospital based and practice based orthopedic care play an important role in the concept of access to orthopedic care. For example, positive effects on access to orthopedic follow-up care were shown by the implementation of a ‘Fracture Care Program’, which aimed at improving the coordination between both health care sectors [54]. Finally, as outlined by Canizares et al., geographic availability of orthopedic surgeries is closely related to accessibility of GPs [25]. Therefore, further research should also take accessibility of GPs into account.

### Conclusion

Limited resources force health care planners to provide care where it is most needed. Despite the advantages of simpler measures regarding implementation and acceptance in health policy, more sophisticated measures have the potential to reduce costs as well as improve health care. With this study, significant geographical variations were revealed that show the need to reduce oversupply in less deprived urban areas in order to enable adequate care in more deprived rural areas. Especially for orthopedic care, accessibility is a major issue. Therefore, health care planners should consider geospatial techniques as presented within this article in order to optimize resource allocation.

## Supporting information

S1 Appendix Detailed results for all 266 regions in Germany according to the definition by the Federal Office for Building and Regional Planning. (XLSX)

S2 Appendix Detailed results for all 385 planning regions as defined by the National Association of Statutory Health Insurance Physicians. (XLSX)
